# Relationship between the laboratory test-based frailty index and overall mortality in critically ill patients with acute pancreatitis: a retrospective study based on the MIMIC-IV database

**DOI:** 10.3389/fmed.2025.1524358

**Published:** 2025-04-08

**Authors:** Li Jin, Yan-Yan Dong, Jun-Peng Xu, Mao-Sheng Chen, Rui-Xiang Zeng, Li-Heng Guo

**Affiliations:** ^1^The Second Clinical College of Guangzhou University of Chinese Medicine, Guangzhou, China; ^2^Department of Critical Care Medicine, Guangdong Provincial Hospital of Chinese Medicine, Guangzhou, China

**Keywords:** acute pancreatitis, frailty, frailty index, laboratory tests, MIMIC-IV database, mortality

## Abstract

**Background and aims:**

The frailty index, based on laboratory assessments, helps identify individuals at risk for adverse health outcomes. However, its relationship with overall mortality in acute pancreatitis patients in ICUs remains unclear. This study aims to investigate the association between the frailty index and all-cause mortality and assess its prognostic value for these patients.

**Methods:**

We carried out a retrospective observational investigation utilizing data from the Medical Information Mart for Intensive Care IV (MIMIC-IV 2.2) database. Extract data from the database for all ICU patients (first-time ICU admissions, age ≥ 18 years) who meet the diagnostic criteria for acute pancreatitis. The frailty index derived from laboratory tests (FI-lab) encompassed three vital sign indicators and 30 laboratory test indicators. Patients were categorized into four groups based on quartiles of the FI-lab score. To assess the differences in 28-day all-cause mortality among these groups, we employed Kaplan–Meier analysis, whereas the relationship between FI-lab scores and 28-day mortality was explored through Cox proportional hazards analysis. In addition, we applied Harrell’s C statistic, Integrated Discrimination Improvement (IDI), and Net Reclassification Improvement (NRI) to assess the additional predictive capability of FI-lab scores compare to traditional disease severity metrics.

**Results:**

The study included a total of 741 patients (all age ≥ 18 years, 19.84% age > 75 years, 41.16% Female). The Kaplan–Meier analysis demonstrated that individuals with elevated FI-lab scores exhibited a significantly heightened risk of all-cause mortality (log-rank *p* < 0.0001). The multivariate Cox regression analysis suggested that treating FI-lab as a continuous variable (per 0.01 increment) was linked to an increased risk of 28-day all-cause mortality [hazard ratio (HR) 1.072, 95% confidence interval (CI) (1.055–1.089), *p* < 0.001]. Moreover, when FI-lab was analyzed as a categorical variable, patients in the fourth quartile of FI-lab had a notably greater risk of 28-day all-cause mortality in comparison to those in the first quartile [HR 9.933, 95% CI (4.676–21.104), *p* < 0.001]. Additionally, the integration of FI-lab scores with conventional disease severity scores improved the predictive performance for 28-day mortality.

**Conclusion:**

In patients in the ICU who have been diagnosed with acute pancreatitis, the FI-lab score functions as a reliable indicator of short-term mortality. Early detection of patients at high risk for acute pancreatitis through the implementation of the FI-lab score, along with prompt interventions, is essential for enhancing these individuals’ prognoses.

## 1 Introduction

Frailty, a multisystem functional decline that increases vulnerability to stressors, has emerged as a critical global health challenge (1, 2). Clinically significant correlations exist between frailty and elevated risks of hospitalization, prolonged hospital stays, and increased healthcare expenditures (3, 4). More critically, frailty independently predicts adverse outcomes including reduced quality of life and higher mortality rates (5).

Acute pancreatitis (AP) is one of the common gastrointestinal diseases encountered in intensive care units (ICUs) (6). It is projected that by 2050, the population of individuals aged 65 and above suffering from pancreatitis may increase by nearly 66% (7). Patients with severe conditions require longer hospital stays and ICU monitoring and treatment, which can even be life-threatening (6, 8). Although severity assessment tools such as the Acute Physiology and Chronic Health Evaluation II (APACHE II) score, Ranson’s criteria for acute pancreatitis, and the Bedside Index for Severity in Acute Pancreatitis (BISAP) score are available, these tools only provide moderate predictive value for mortality and clinical outcomes in hospitalized patients (9, 10). Frailty is highly prevalent among ICU patients with acute pancreatitis. In the population with gastrointestinal diseases, frailty is associated with poor prognosis and higher complication rates in patients with chronic pancreatitis, common bile duct stones, or cirrhosis (11, 12). For patients with acute pancreatitis, there is a paucity of research on frailty, and clinical evidence is insufficient. Currently, there is no unified method for assessing the degree of frailty in patients with acute pancreatitis. It is crucial to evaluate the impact of frailty on the prognosis of patients with acute pancreatitis and to select appropriate assessment tools, which warrants further in-depth investigation. This study is the first to investigate the relationship between the FI-lab and the prognosis of ICU patients with acute pancreatitis.

Commonly used frailty assessment tools include the Clinical Frailty Scale (CFS), the Hospital Frailty Risk Score (HFRS), and the frailty index based on laboratory values (FI-lab). The selection of a frailty assessment tool should be based on research and clinical needs and objectives. The Clinical Frailty Scale (CFS) (13) primarily relies on clinical judgment, determining a patient’s frailty level through inquiries with the patient or their family members, combined with information on the patient’s medical history, clinical presentation, and daily activity capabilities. Its limitation lies in the need for prior training of clinicians, as assessment results tend to be subjective and susceptible to personal biases (14). The Hospital Frailty Risk Score (HFRS) (15) evaluates a patient’s frailty risk based on their current and past ICD-10 codes. The HFRS also has limitations, it requires prior medical data of the patient and may be constrained by incomplete or potentially inaccurate data, as well as the inability of ICD-10 codes to reflect disease severity, since past diagnostic records may have been used solely for reimbursement purposes (16).

FI-lab was proposed by Howlett et al. (17). Compared to the CFS and HFRS, its innovations and advantages include (18): (a) reducing the influence of subjective judgments on assessment results, exhibiting strong objectivity; (b) not requiring complex clinical assessments or questionnaires, making the assessment process simple; and (c) being applicable to various clinical settings, with a scope of application no less than that of the CFS and HFRS. In ICU patients, incomplete medical histories, difficulty in physical examination cooperation, or inability to effectively communicate are common. Affected by these factors, the assessment results of the CFS and HFRS may not be accurate enough, making the FI-lab more suitable for assessing frailty in this population. Studies have demonstrated that the FI-lab is a reliable tool for predicting adverse outcomes such as mortality, increased medication use, and frequency of medical visits (19–21). Among patients admitted to the ICU, the FI-lab has been associated with all-cause short-term and long-term mortality in conditions such as septic shock, chronic heart failure, and acute kidney injury (18, 22, 23). Currently, no studies have explored the relationship between frailty levels and the FI-lab in ICU patients with acute pancreatitis, highlighting the necessity for further research to assess the significance of the FI-lab in this patient population.

This research aims to investigate the relationship between FI-lab results and mortality rates after 28 days in ICU patients diagnosed with acute pancreatitis. Data for this study were sourced from the Medical Information Mart for Intensive Care IV (MIMIC-IV) version 2.2 database. Furthermore, we evaluate the added predictive capability of integrating FI-lab into the well-established disease severity scoring systems, which consist of the Sequential Organ Failure Assessment (SOFA), Acute Physiology Score III (APS3), Simplified Acute Physiology Score II (SAPS2), Logistic Organ Dysfunction System (LODS), and Oxford Acute Severity of Illness Score (OASIS).

## 2 Methods

### 2.1 Data source and study population

This research constitutes a retrospective observational study, aimed at examining the data of critically ill patients. The patient information was meticulously gathered from the Medical Information Mart for Intensive Care IV (MIMIC-IV) database, which is a publicly accessible repository that houses comprehensive data regarding patients who were admitted to the intensive care units at Beth Israel Deaconess Medical Center (BIDMC) in Boston, United States, over a span from 2008 to 2019. During data collection, our team ensured strict compliance with all pertinent regulations to uphold ethical standards. Author Li Jin successfully obtained the Collaborative Institutional Training Initiative (CITI) certificate, identified by Record ID 44240625, which is a prerequisite for conducting research that involves human participants. Furthermore, we secured the necessary approvals to utilize the MIMIC-IV database for data extraction purposes. The project gained formal approval from both the Institutional Review Board of the Massachusetts Institute of Technology and BIDMC, underscoring its commitment to ethical research standards. The entire study was conducted in a systematic manner in accordance with the principles specified in the Strengthening the Reporting of Observational Studies in Epidemiology-Cohort, Cross-Sectional, and Case–Control Studies (STROCSS), thereby guaranteeing a robust standard of rigor in our results.

For the purpose of analysis, we exclusively included patients admitted to ICU for the first time with a confirmed diagnosis of acute pancreatitis (obtained through visual inspection of the MIMIC-IV database). To ensure data completeness and reliability, patients meeting any of the following criteria were excluded: (a) age < 18 years; (b) ICU length of stay <24 h; (c) excessive missing items (*n* > 6) in the FI-lab scale components. These exclusion criteria were essential to preserve the validity of FI-lab assessments. This study specifically focused on adult populations, as pediatric patients were excluded to minimize potential confounding effects arising from physiological, metabolic, and pharmacological differences between minors and adults.

The calculation of FI-lab scores required a minimum 24-h observation period to ensure data availability and integrity (17). Excessive missing items (>6) in the FI-lab scale construction were deemed unacceptable based on previous comparable studies, as they would compromise scoring accuracy and validity (22, 23). Consequently, we excluded patients with ICU stays <24 h and those with >6 missing scale items. While the exclusion of patients with short ICU stays and missing critical variables might introduce selection bias, these measures were considered necessary to ensure the robustness and reliability of study findings. Future investigations with larger sample sizes and more comprehensive datasets may help mitigate this potential bias.

**Figure 1:**
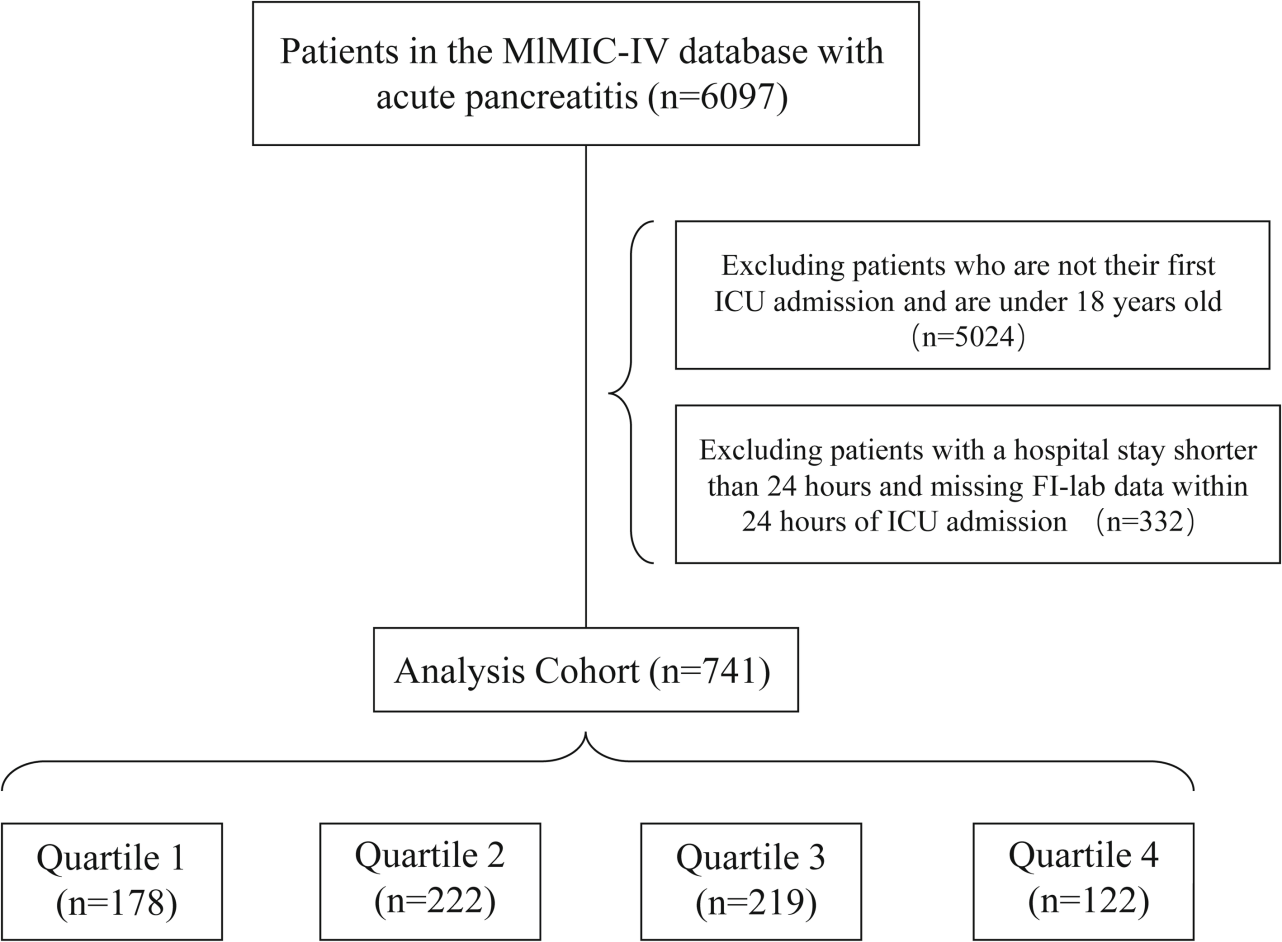
Flow chart of the study.

Seven hundred and forty-one acute pancreatitis patients (all age ≥ 18, 19.84% age > 75, 41.16% female, 60.86% white) were ultimately enrolled. The included patients were stratified into distinct groups based on quartiles of their initial FI-lab scores, which were assessed during the first 24 h following ICU admission. A visual representation of the patient selection process is provided in Figure 1 for enhanced clarity.

### 2.2 The establishment of FI-lab

The FI-lab score utilizes a total of 33 variables, including 3 vital signs and 30 laboratory indicators. These variables were retained in alignment with established FI-lab construction criteria and prior studies on FI-lab scoring in critically ill populations (17, 22). The specific components, reference ranges, and detailed descriptions of all 33 variables are provided in Supplementary Table S1. Each variable was standardized according to its reference range. For all parameters, a value of 1 was assigned if results exceeded the normal range, while a value of 0 was assigned for values within the reference range. The FI-lab score was computed by summing all assigned values and dividing by the total number of variables assessed. For example: (a) A hemoglobin level of 10 g/dL (below the reference range of 12–16 g/dL) would yield a value of 1 for this variable; (b) A creatinine level of 1.2 mg/dL (within the reference range of 0.5–1.2 mg/dL) would be assigned a value of 0. Patients were subsequently stratified into quartiles based on their FI-lab scores for further statistical analysis.

### 2.3 Covariate extraction

Data regarding the baseline characteristics of patients during their current hospitalization were obtained from the MIMIC-IV database. This comprehensive dataset encompassed various demographic and clinical variables, including gender, age, race, and body mass index (BMI). Additionally, key scoring systems that assess organ dysfunction were utilized, such as the Oxford Acute Severity of Illness Score (OASIS), the Sequential Organ Failure Assessment (SOFA), and the Simplified Acute Physiology Score II (SAPS2). Moreover, the Acute Physiology Score III (APS3) and the Logistic Organ Dysfunction System (LODS) were also incorporated. The interventions applied in the study encompassed essential actions like mechanical ventilation, continuous renal replacement therapy (CRRT), sedation administration, the use of vasoactive drugs, and insulin therapy. Such treatments are vital for the care of patients experiencing severe acute illnesses. Furthermore, the study documented a range of comorbidities that patients might have been suffering from, including coronary artery disease (CAD), heart failure (HF), liver disease, liver cirrhosis, diabetes, renal disease, chronic obstructive pulmonary disease (COPD), and malignancy. Additional conditions like cardiogenic shock, septic shock, and stroke were also recorded, with all comorbidities classified according to the International Classification of Diseases, Tenth Edition (ICD-10) and Ninth Edition (ICD-9). To address missing data in the dataset, the Multiple Imputation by Chained Equations (MICE) method was implemented. This approach involves generating multiple imputed datasets, analyzing each dataset separately, and subsequently pooling the results to produce consolidated estimates. For included patients, missing values (≤15% per variable) were imputed (5 imputations, 10 iterations). The imputation models included predictive mean matching for continuous variables and logistic regression for categorical variables. The analysis was predicated on the assumption that data were missing at random (MAR), implying that the probability of missingness depended solely on observed data and not on unobserved values. By implementing these methodologies, the study aimed to mitigate potential biases that could arise from incomplete datasets while ensuring a thorough examination of the patient characteristics and treatment outcomes.

### 2.4 Statistical analysis

In this study, continuous variables were assessed using two distinct statistical methods: the mean accompanied by the standard deviation (SD) and the median paired with the interquartile range (IQR). The selection of these statistical measures was influenced by the specific characteristics of the data being analyzed. Consequently, based on these characteristics, appropriate methods for group comparisons were employed, utilizing either the Mann–Whitney *U* test or Student’s *t*-test to ensure accurate and relevant statistical analysis. For categorical variables, the data were represented as frequencies and percentages. When examining differences between groups for these variables, specific tests such as Fisher’s exact test or Pearson’s chi-square test were utilized. The FI-lab data were multiplied by 100 for statistical analysis.

Utilizing the FI-lab, the Kaplan–Meier survival analysis was performed to analyze the incidence of primary outcome events in different stratified groups. The log-rank test was applied to assess the observed differences. Additionally, Cox proportional hazards models were used to calculate the hazard ratio (HR) and 95% confidence interval (CI) for 28-day mortality risk in acute pancreatitis patients, incorporating FI-lab and multivariable analysis. Model 1 was designed to focus solely on the FI-lab score, providing a baseline evaluation without additional adjustments. In contrast, Model 2 introduced essential modifications to account for important demographic and clinical factors, specifically age, gender, race, and BMI. Model 3 included the variables from Model 2 while additionally adjusting for other factors such as mechanical ventilation usage, CRRT, sedatives, vasoactive drugs, insulin, HF, CAD, stroke, diabetes, renal disease, liver disease, COPD, malignancy, cardiogenic shock, septic shock, and liver cirrhosis. In all the models utilized, the reference category was established as the lowest quartile of the FI-lab. The evaluation of the proportional hazards assumption was carried out using Schoenfeld residuals. In order to examine the relationship between dose and response, as well as the risk associated with the primary outcome, we employed restricted cubic spline analysis. Additionally, we performed stratified analyses to determine how consistently FI-lab’s prognostic value correlates with the primary outcome in various subgroups. Covariates for subgroup analysis—including sex, age (≤75 vs. >75 years), race, BMI (<30 vs. ≥30 kg/m^2^), and comorbidities—were selected based on clinical relevance and their potential impact on patient outcomes. BMI has been demonstrated to correlate with diverse health outcomes, including mortality. Comorbidities such as diabetes, renal disease, liver disease, and malignancy were included due to their significant prognostic influence on critically ill patients, particularly in severe cases. In Cox regression models, the proportional hazards assumption was evaluated using Schoenfeld residual tests, with no significant deviations observed (*p* > 0.05 for all covariates). This assumption posits that hazard ratios remain constant over time, implying that predictor effects on risk do not vary temporally. Violations of this assumption may lead to biased estimates.

FI-lab was incorporated into existing disease severity scores (SOFA score, LODS score, OASIS score, APS3 score, and SAPS2 score) to evaluate whether it could improve the prediction accuracy for adverse outcome events. Discrimination metrics, specifically Harrell’s C statistic, were utilized to evaluate the predictive efficacy of the models. The DeLong test was utilized to evaluate the C statistics of models incorporating FI-lab against those that excluded it. To determine the additional predictive value of including FI-lab in the disease severity scoring systems, we computed the C statistic in conjunction with the integrated discrimination improvement (IDI) and net reclassification improvement (NRI). Additionally, we conducted Decision Curve Analysis (DCA) to demonstrate the net benefit of the FI-lab combined model across risk thresholds.

Data analysis was conducted utilizing R software (version 4.3.1). A two-tailed *p*-value of less than 0.05 was deemed statistically significant for all analyses.

## 3 Results

### 3.1 Baseline characteristics

Table 1 illustrates the primary characteristics categorized by FI-lab quartiles. The overall median FI-lab was 0.48. The mean (SD) for the four FI-lab groups were 0.32 (0.06), 0.45 (0.03), 0.55 (0.03), and 0.69 (0.06), respectively. Compared with the Q1 group, patients in the Q4 group had higher disease severity scores at admission (*p* < 0.001), higher rates of mechanical ventilation, CRRT treatment, sedative use, vasoactive drug use, and insulin use, as well as higher prevalence of renal disease, liver disease, cardiogenic shock, and septic shock. The analysis revealed that there were no statistically significant differences in terms of gender, body mass index (BMI), or race among the four groups studied. However, notable distinctions were observed in the demographic characteristics of the Q3 group, which comprised older and heavier patients compared to the other groups, with a significance level of *p* < 0.05. Furthermore, the 28-day mortality rate exhibited a concerning upward trend correlating with the increase in FI-lab scores. Specifically, the mortality rates for each group were as follows: Q1 recorded a rate of 4.49%, Q2 had a rate of 6.31%, Q3 showed a significantly higher rate of 16.44%, and Q4 revealed a staggering rate of 36.07% (*p* < 0.001).

**Table 1:** Basic demographic characteristics of the original cohort.

Variables	Q1 (*N* = 178)	Q2 (*N* = 222)	Q3 (*N* = 219)	Q4 (*N* = 122)	*p*-value	Missing data (%)
Events						
28 day mortality	**8 (4.49%)**	**14 (6.31%)**	**36 (16.44%)**	**44 (36.07%)**	**<0.001**	**0.0**
Demographic						
Age	**57.10 (17.33)**	**56.37 (17.97)**	**61.69 (16.88)**	**59.45 (16.46)**	**<0.01**	**0.0**
Gender						
Male	107 (60.11%)	134 (60.36%)	125 (50.08%)	70 (57.38%)	0.869	0.0
Female	71 (39.89%)	88 (39.64%)	94 (42.92%)	52 (42.62%)		
Weight	**82.25 (23.58)**	**88.38 (23.65)**	**86.89 (21.97)**	**86.75 (29.48)**	**<0.05**	**0.4**
Height	170.22 (7.19)	170.49 (7.24)	169.18 (8.20)	168.64 (8.54)	0.157	0.0
BMI	29.09 (5.11)	30.02 (6.41)	30.33 (5.56)	30.87 (13.82)	0.241	0.0
Race						
White	107 (60.11%)	151 (68.02%)	123 (56.16%)	70 (57.38%)	0.188	0.0
Asian	6 (3.37%)	6 (2.70%)	8 (3.65%)	6 (4.92%)		
Black	27 (15.17%)	20 (9.01%)	23 (10.50%)	14 (11.48%)		
Other	38 (21.35%)	45 (20.27%)	65 (29.68%)	32 (26.23%)		
Disease severity scoring system						
SAPSII	**28.96 (11.66)**	**34.42 (13.90)**	**42.54 (15.51)**	**55.30 (15.40)**	**<0.001**	**0.0**
SOFA	**3.68 (2.76)**	**5.39 (3.33)**	**7.53 (3.67)**	**11.36 (4.00)**	**<0.001**	**0.0**
APSIII	**38.70 (16.48)**	**49.37 (17.40)**	**61.53 (21.61)**	**84.07 (24.79)**	**<0.001**	**0.0**
OASIS	**30.19 (7.56)**	**33.01 (8.06)**	**35.92 (8.71)**	**42.58 (8.62)**	**<0.001**	**0.0**
LODS	**3.45 (2.41)**	**4.83 (2.77)**	**6.17 (3.04)**	**8.85 (3.20)**	**<0.001**	**0.0**
Interventions						
Mechanical ventilation	**60 (33.71%)**	**96 (43.24%)**	**112 (51.14%)**	**92 (75.41%)**	**<0.001**	**0.0**
CRRT	**1 (0.56%)**	**3 (1.35%)**	**15 (6.85%)**	**21 (17.21%)**	**<0.001**	**0.0**
Sedative	**68 (38.20%)**	**108 (48.65%)**	**124 (56.62%)**	**90 (73.77%)**	**<0.001**	**0.0**
Vasopressor	**33 (18.54%)**	**57 (25.68%)**	**82 (37.44%)**	**82 (67.21%)**	**<0.001**	**0.0**
Insulin	**54 (30.34%)**	**78 (35.14%)**	**96 (43.84%)**	**58 (47.54%)**	**<0.01**	**0.0**
Comorbidities						
HF	34 (19.10%)	47 (21.17%)	31 (14.16%)	24 (19.67%)	0.266	0.0
CAD	27 (15.17%)	37 (16.67%)	36 (16.44%)	21 (17.21%)	0.966	0.0
Stroke	6 (3.37%)	5 (2.25%)	8 (3.65%)	8 (6.56%)	0.239	0.0
Diabetes	45 (25.28%)	70 (31.53%)	73 (33.33%)	35 (28.69%)	0.336	0.0
Renal	**20 (11.24%)**	**33 (14.86%)**	**48 (21.92%)**	**28 (22.95%)**	**<0.01**	**0.0**
Liver	**26 (14.61%)**	**34 (15.32%)**	**57 (26.03%)**	**30 (24.59%)**	**<0.01**	**0.0**
Liver Cirrhosis	**15 (8.43%)**	**26 (11.71%)**	**31 (14.16%)**	**22 (18.03%)**	**0.083**	**0.0**
COPD	23 (12.92%)	30 (13.51%)	26 (11.87%)	16 (13.11%)	0.963	0.0
Malignancy	19 (10.67%)	32 (14.41%)	35 (15.98%)	17 (13.93%)	0.496	0.0
Cardiogenic shock	**4 (2.25%)**	**5 (2.25%)**	**8 (3.65%)**	**10 (8.20%)**	**<0.05**	**0.0**
Septic shock	**20 (11.24%)**	**38 (17.12%)**	**53 (24.20%)**	**65 (53.28%)**	**<0.001**	**0.0**
Laboratory tests (1st 24 h)						
FI-lab	**0.32 (0.06)**	**0.45 (0.03)**	**0.55 (0.03)**	**0.69 (0.06)**	**<0.001**	**0.0**

This table presents the baseline characteristics of the study population, categorized by quartiles of the FI-lab score. Variables include demographic information, disease severity scores, interventions, comorbidities, and laboratory tests. *p*-values indicate statistical significance between quartiles. Quartile ranges for the FI-lab are as follows: Q1: 0.07–0.41; Q2: 0.41–0.48; Q3: 0.48–0.59; and Q4: 0.59–0.85.

Abbreviations include body mass index (BMI); Simplified Acute Physiology Score II (SAPS2); Sequential Organ Failure Assessment (SOFA); Acute Physiology Score III (APS3); Oxford Acute Severity of Illness Score (OASIS); Logistic Organ Dysfunction System (LODS); continuous renal replacement therapy (CRRT); heart failure (HF); coronary artery disease (CAD); chronic obstructive pulmonary disease (COPD); and frailty index determined by laboratory tests (FI-lab).

Statistical Tests: continuous variables are presented as mean (SD) and compared using ANOVA or Kruskal–Wallis test as appropriate. Categorical variables are presented as *n* (%) and compared using chi-square or Fisher’s exact test. The values are expressed as mean (with standard deviation) for continuous variables and as number (with percentage) for categorical variables. Variables in bold have *p*-value < 0.05.

### 3.2 All-cause mortality rate

**Figure 2:**
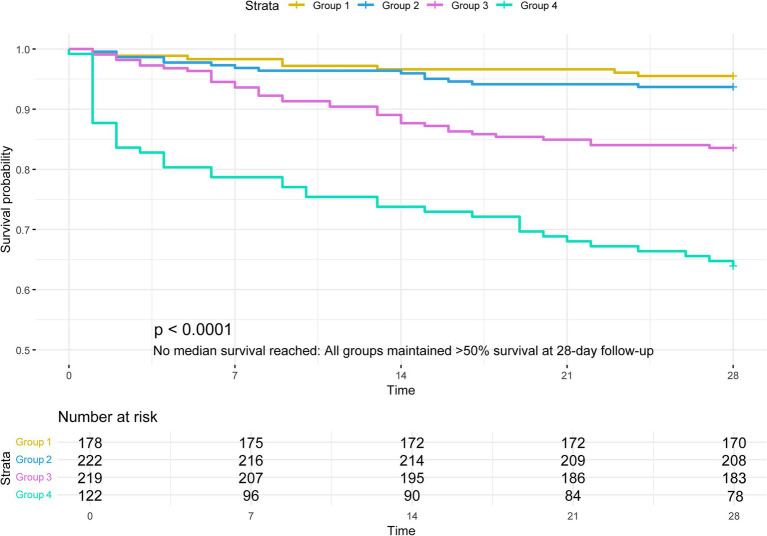
Kaplan–Meier survival analysis curves for all-cause mortality.

The Kaplan–Meier survival curves in Figure 2 demonstrate the all-cause mortality rates for each group based on FI-lab quartiles. During the follow-up period, the groups exhibiting elevated FI-lab values experienced increased mortality rates at 28 days (Q1: 4.49% compared to Q2: 6.31%, Q3: 16.44%, and Q4: 36.07%; log-rank *p* < 0.0001). The results demonstrated that the Q4 group (patients with the highest FI-lab scores) exhibited a statistically significant increase in mortality compared to the Q1 group (patients with the lowest FI-lab scores). Higher FI-lab scores reflect greater frailty severity, suggesting more pronounced physiological decline in the Q4 cohort. This compromised physiological reserve likely reduced their capacity to mount effective stress responses to acute pancreatitis, thereby elevating risks of complications and mortality. These findings are corroborated by the Q4 group’s elevated rates of mechanical ventilation, CRRT utilization, vasoactive agent administration, and comorbidity prevalence, as detailed in Table 1.

### 3.3 Association between all-cause mortality risk and FI-lab

**Figure 3:**
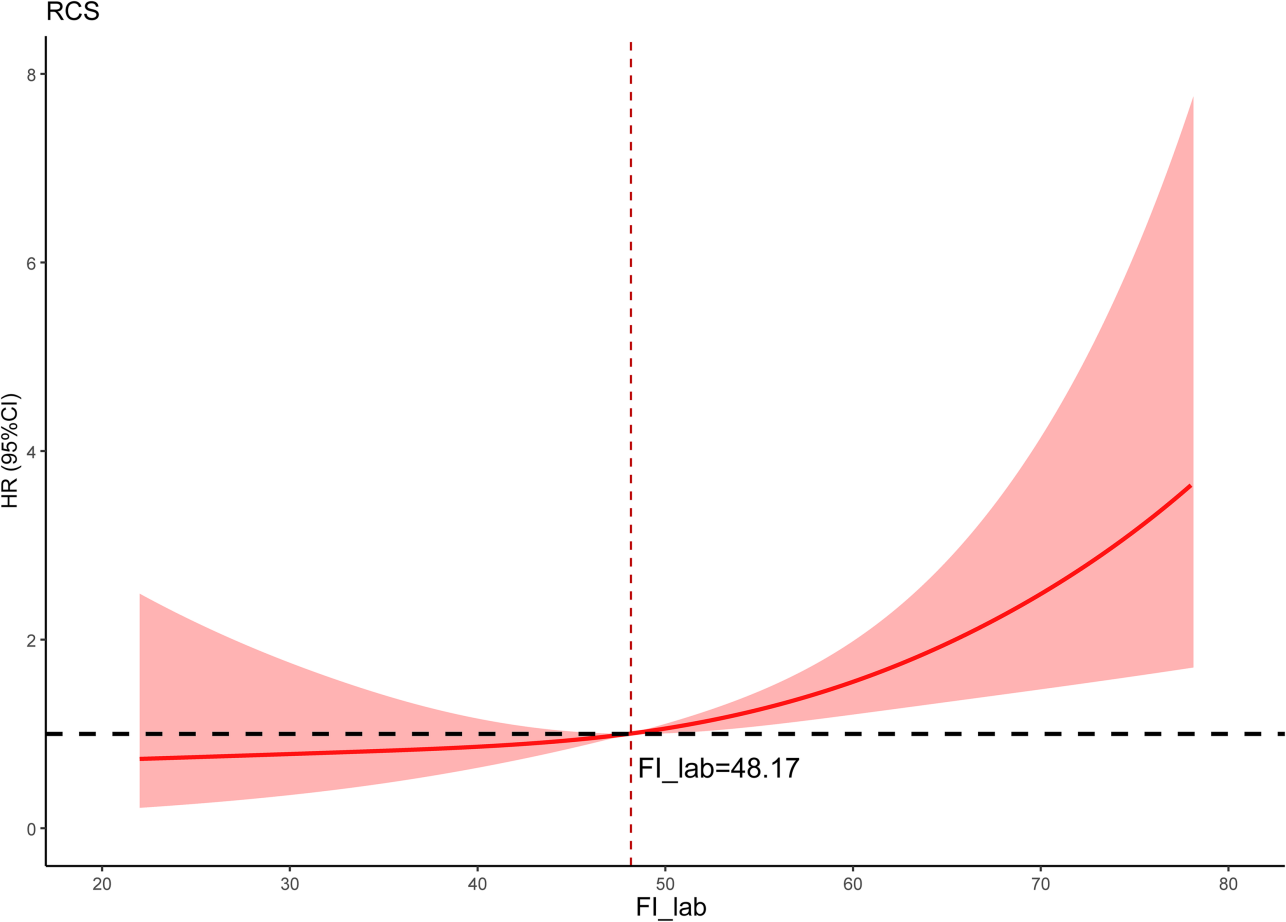
Restricted cubic spline regression analysis of FI-lab with all-cause mortality.

When treated as a continuous variable, Cox proportional hazards analysis revealed a significant association between FI-lab and 28-day mortality in both Model 1 [HR 1.072, 95% CI (1.055–1.089), *p* < 0.001 per 0.01-score increase] and Model 3 [HR 1.036, 95% CI (1.014–1.058), *p* < 0.05 per 0.01-score increase]. When treated as a continuous variable in Model 3, the 28-day mortality risk was higher in the Q4 group compared to the Q1 group, and it increased with higher FI-lab scores [Q1 vs. Q4: HR 3.011, 95% CI (1.214–7.470), *P* for trend <0.05] (Table 2). To enhance the credibility of the results, we conducted internal validation of Model 3 using Bootstrap resampling (1,000 iterations). The results showed that the mean C-statistic was 0.889 (SD = 0.013, 95% CI: 0.863–0.914), demonstrating the stability of the results. Furthermore, the restricted cubic spline regression model demonstrated a linear increase in the risk of 28-day mortality with increasing FI-lab scores (Figure 3).

**Table 2:** Cox proportional hazard ratio (HR) for all-cause mortality.

Variables	Model 1			Model 2			Model 3		
	HR (95% CI)	*P*	*P* for trend	HR (95% CI)	*P*	*P* for trend	HR (95% CI)	*P*	*P* for trend
28 day death									
Per 0.01 unit	1.072 (1.055–1.089)	**<0.001**		1.072 (1.054–1.089)	**<0.001**		1.036 (1.014–1.058)	**0.009**	
Quartile									
Q1	Ref.		**<0.001**	Ref.		**<0.001**	Ref.		**0.01**
Q2	1.417 (0.595–3.378)	0.431		1.430 (0.600–3.412)	0.42		1.097 (0.438–2.745)	0.844	
Q3	3.871 (1.799–8.329)	**<0.001**		3.166 (1.468–6.826)	**0.003**		1.744 (0.762–3.992)	0.222	
Q4	9.933 (4.676–21.104)	**<0.001**		9.351 (4.391–19.914)	**<0.001**		3.019 (1.213–7.513)	**0.017**	

This table presents the results of the Cox proportional hazards regression model, which was used to assess the association between the FI-lab score and 28-day all-cause mortality. The Cox proportional hazards regression model was employed to adjust for potential confounding factors, allowing for a more accurate evaluation of the relationship between the FI-lab score and mortality. As the FI-lab score increases, the risk of 28-day all-cause mortality among patients significantly rises.

Model 1: unadjusted. Model 2: adjusted for age, gender, race, and BMI. Model 3: adjusted for age, gender, race, and BMI, use of mechanical ventilation, CRRT, sedatives, vasoactive drugs, insulin, HF, CAD, stroke, diabetes, renal disease, liver disease, COPD, malignancy, cardiogenic shock, septic shock, and liver cirrhosis. Variables in bold have *p*-value < 0.05.

**Figure 4:**
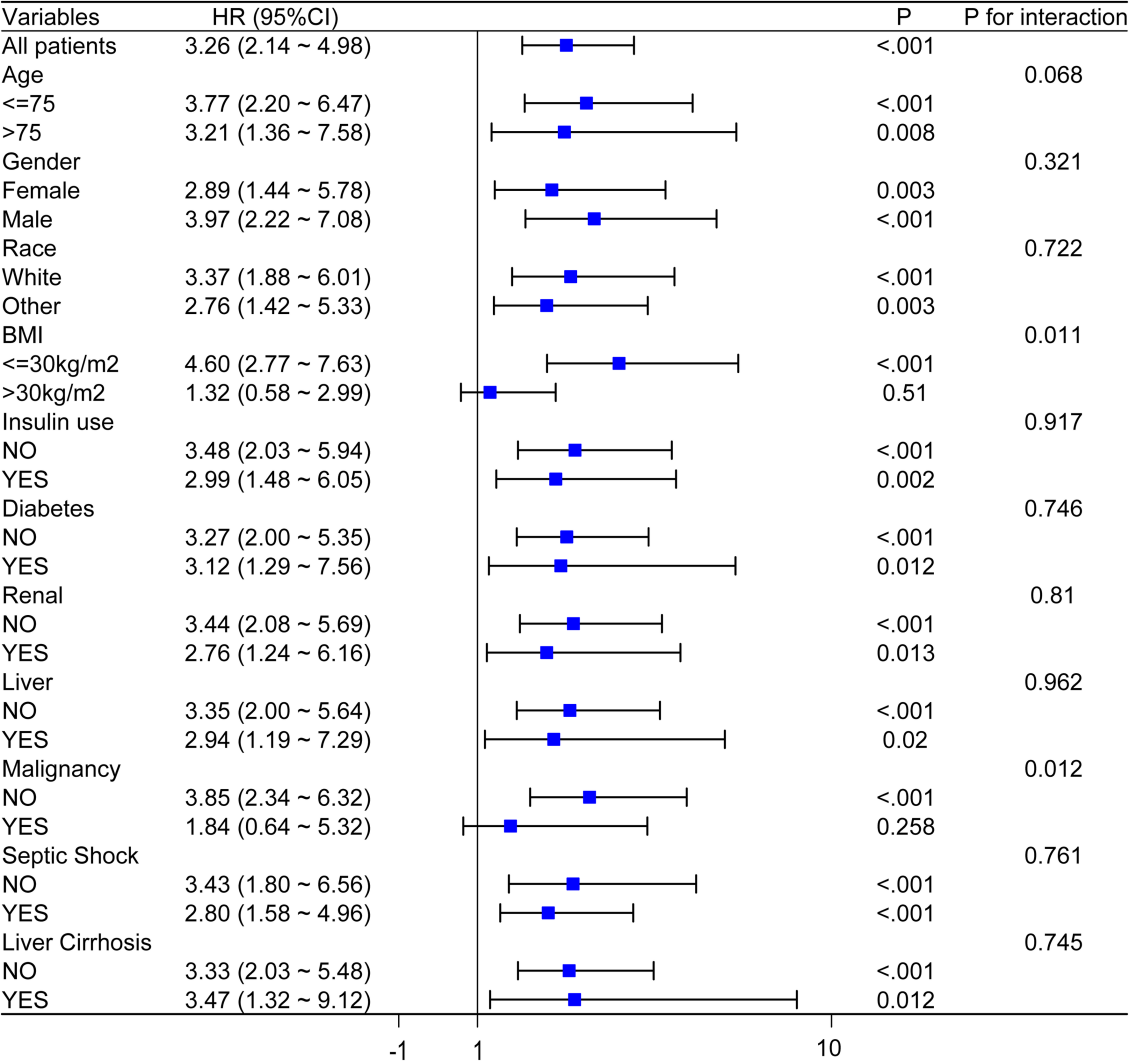
Forest plots of hazard ratios (HRs) for the 28 day all-cause mortality in different subgroups.

Stratified analysis of the relationship between FI-lab and 28-day all-cause mortality was conducted based on factors such as age, gender, race, BMI, insulin use, diabetes, renal disease, liver disease, septic shock, and cirrhosis. After excluding subgroups with BMI > 30 kg/m^2^ and those with malignancy, the FI-lab was significantly associated with the 28-day mortality risk in the remaining subgroups (all *p* < 0.05). Furthermore, the predictive value of FI-lab was even more pronounced in the subgroup with BMI ≤ 30 kg/m^2^ and malignancy, as illustrated in Figure 4. Obesity is a well-established risk factor for acute pancreatitis (24), which may influence clinical outcomes in affected patients. After adjusting for confounding factors including obesity, the FI-lab score retained prognostic utility in non-obese populations. This suggests that the degree of physiological decline captured by the FI-lab score independently contributes to prognosis in acute pancreatitis, beyond the effects of obesity. Patients with malignancy frequently exhibit accelerated physiological decline and compromised immune function, resulting in heightened systemic vulnerability. Consequently, the FI-lab score may more precisely identify prognostically adverse physiological alterations in this population. These findings support the integration of BMI, malignancy status, and FI-lab scores in clinical practice to enable comprehensive risk stratification and personalized therapeutic strategies for acute pancreatitis patients.

### 3.4 Incremental value of FI-lab for 28 day mortality

**Figure 5:**
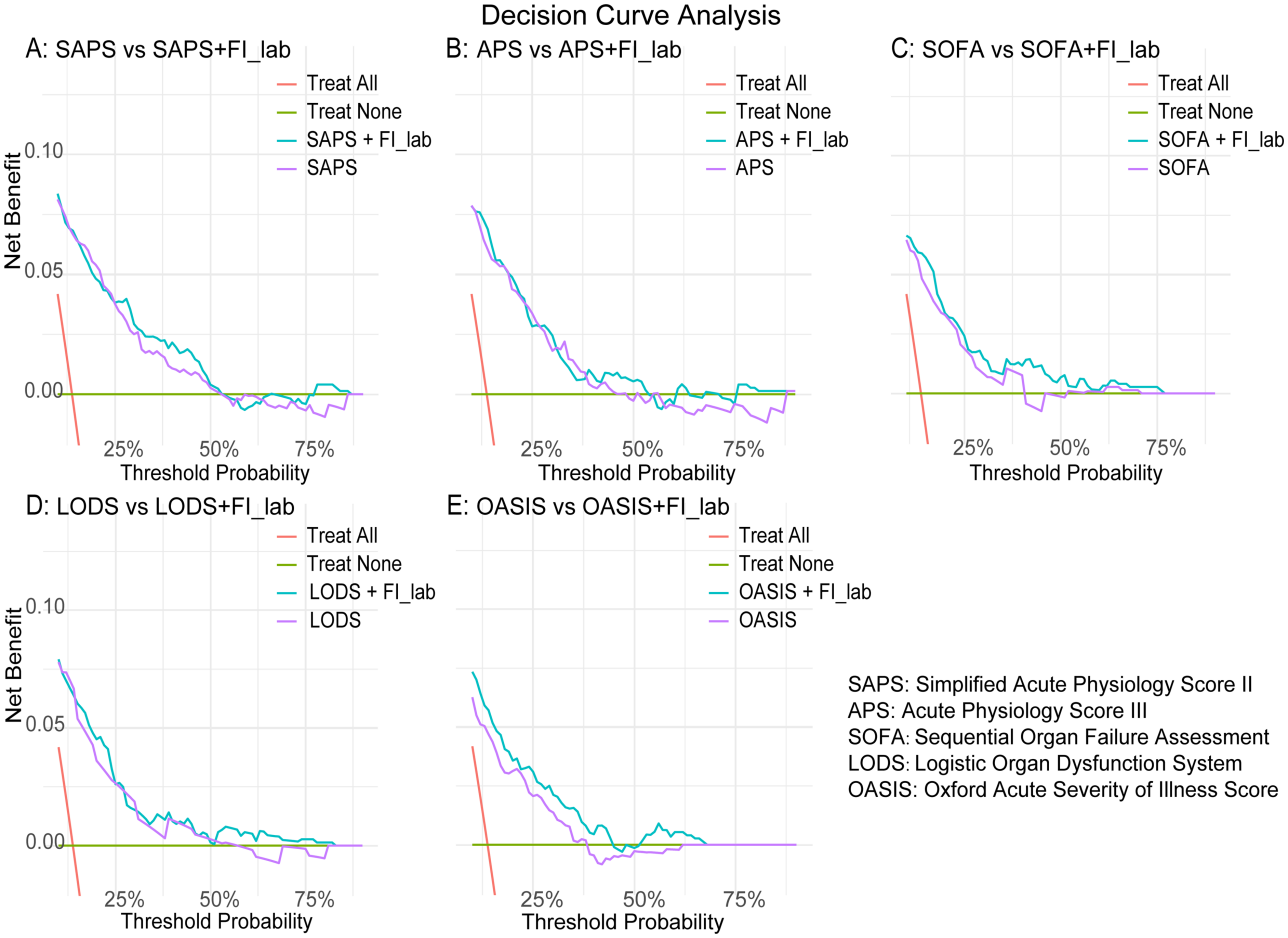
Decision curve analysis. **(A)** SAPS vs. SAPS + FI-lab; **(B)** APS vs. APS + FI-lab; **(C)** SOFA vs. SOFA + FI-lab; **(D)** LODS vs. LODS + FI-lab; **(E)** OASIS vs. OASIS + FI-lab.

We evaluated the ability of five disease severity scoring systems (including SAPS2, APS3, SOFA, LODS, and OASIS) to predict 28-day mortality. The results showed that the C-statistic was applicable to all systems, ranging from 0.715 to 0.812. Incorporating FI-lab into each scoring system improved the discriminatory ability of all scores, with Integrated Discrimination Improvement (IDI) values of 0.027, 0.028, 0.038, 0.034, and 0.062 for SAPS2, APS3, SOFA, LODS, and OASIS, respectively (Table 3). The Net Reclassification Improvement (NRI) significantly increased when predicting probabilities (NRI values for SAPS2, APS3, SOFA, LODS, and OASIS were 0.487, 0.559, 0.512, 0.428, and 0.529, respectively; Table 3). The results of Decision Curve Analysis demonstrate that, across a wide range of threshold probabilities (10–50%), the FI-lab combined models (OASIS + FI-lab and SOFA + FI-lab) significantly improve the clinical net benefit compared to single scores (Figure 5).

**Table 3:** Incremental value of FI-lab for 28 day mortality.

Models	C statistic^a^	C statistic^b^	ΔC statistic	IDI (95% CI)	NRI (95% CI)
28 day mortality					
SAPS2 vs. SAPS2 + FI-lab	0.818 (0.781–0.854)	0.812 (0.775–0.850)	0.005	0.027 (0.004–0.071)	0.487 (0.265–0.687)
APS3 vs. APS3 + FI-lab	0.798 (0.758–0.838)	0.798 (0.760–0.837)	0	0.028 (0.004–0.069)	0.559 (0.263–0.773)
SOFA vs. SOFA + FI-lab	0.761 (0.716–0.806)	0.741 (0.696–0.786)	0.02	0.038 (0.011–0.076)	0.512 (0.265–0.700)
LODS vs. LODS + FI-lab	0.792 (0.752–0.831)	0.777 (0.734–0.819)	0.015	0.034 (0.007–0.078)	0.428 (0.174–0.640)
OASIS vs. OASIS + FI-lab	0.761 (0.715–0.807)	0.715 (0.662–0.768)	0.046	0.062 (0.025–0.114)	0.529 (0.328–0.731)

This table evaluates the improvement in the predictive accuracy of 28-day all-cause mortality when the FI-lab score is incorporated into traditional disease severity scoring systems.

Models: these include a model that only includes the disease severity score and a model that includes both the disease severity score and the FI-lab score.

C-statistic: used to assess the discriminatory power of the model, i.e., the model’s ability to distinguish between survivors and non-survivors.

^aModels including FI-lab and disease severity score. bModels only including disease severity score.^

ΔC-statistic: represents the change in the C-statistic after incorporating the FI-lab score, with a positive value indicating an improvement in predictive accuracy.

IDI (95% CI): integrated discrimination improvement, used to quantify the improvement in the model’s predicted probabilities.

NRI (95% CI): net reclassification improvement, used to assess the improvement in the model’s classification of individual risk.

Abbreviations: the Sequential Organ Failure Assessment (SOFA), Acute Physiology Score III (APS3), Simplified Acute Physiology Score II (SAPS2), Logistic Organ Dysfunction System (LODS), Oxford Acute Severity of Illness Score (OASIS).

## 4 Discussion

Our research marks the initial examination of the connection between FI-lab and 28 day all-cause mortality from all causes in ICU patients suffering from acute pancreatitis. The results suggest that, following adjustments for confounding factors, FI-lab shows a strong correlation with 28-day mortality from all causes in this group of patients. By integrating FI-lab into established classic disease severity scores, we observed an enhancement in the predictive capacity of each scoring system regarding mortality.

Frailty is a complex manifestation resulting from an interplay of various factors, potentially linked to several cellular and tissue mechanisms that remain incompletely understood. These mechanisms include loss of protein homeostasis, chronic inflammation, cellular senescence, telomere attrition, stem cell depletion, altered intercellular communication, mitochondrial dysfunction, and dysbiosis (25, 26). The structural and functional damage inflicted by these multiple mechanisms ultimately heightens the risk of inflammatory imbalance and exacerbates infections in patients with pancreatitis. The significance of frailty in the context of pancreatitis is increasingly acknowledged. Prior evidence indicates that frailty correlates with an increased likelihood of negative health results in pancreatitis, making it an important resource for risk assessment and the detection of patients at high risk (18, 27–29).

In critically ill patients, impaired or declining health function is prevalent. Acute pancreatitis patients exhibiting higher levels of frailty demonstrate diminished recovery capabilities, resulting in extended hospital stays and an increased likelihood of mortality. Nonetheless, clinical evidence regarding frailty in acute pancreatitis patients remains scarce, and no studies have yet examined FI-lab in ICU patients with this condition.

FI-lab was first proposed by Howlett et al. (17) for identifying individuals with an increased risk of mortality among community-dwelling older adults. Blodgett et al. (30) subsequently demonstrated that FI-lab is associated with adverse health outcomes in community-dwelling male populations. Following this, several large cohort studies have indicated that FI-lab correlates with mortality risk in both European and Asian populations (31–33). In terms of frailty identification, FI-lab exhibits comparable discriminative ability to other assessment tools (20). For instance, two studies found that both FI-lab and the Clinical Frailty Scale (CFS) are linked to adverse outcomes (34, 35). Clinically, it is most convenient for practitioners to utilize routinely collected data to assess the degree of frailty. Moreover, routine blood tests and physical measurements (such as heart rate) obtained upon admission necessitate minimal patient cooperation. Consequently, in clinical settings, FI-lab serves as a more user-friendly assessment tool compared to other instruments (33). Patients in ICU are often critically ill and exhibit low levels of cooperation, which can complicate clinicians’ efforts to perform accurate physical examinations and communications. Therefore, FI-lab may be particularly well-suited for frailty assessment in ICU patients. Research conducted by Qin, Li, and others has shown that FI-lab is linked to both long-term and short-term mortality rates in critically ill individuals suffering from acute myocardial infarction and heart failure (22, 36). Additionally, Bai et al. (37) found that a higher FI-lab score correlates with an increased risk of acute kidney injury following heart surgery.

The findings of this study demonstrate that the FI-lab score serves as a valuable clinical tool for predicting short-term mortality risk in ICU-admitted acute pancreatitis patients. Building on previous research (19, 20), the FI-lab score may also hold potential for forecasting long-term outcomes, including 1-year survival rates, functional status recovery, and hospital readmission rates. Future implementations could enable automated FI-lab score calculation within electronic health record systems, providing ICU clinicians with real-time risk assessment capabilities. Clinicians could further enhance risk stratification by integrating the FI-lab score with established prognostic indicators such as Sequential Organ Failure Assessment (SOFA) scores, inflammatory biomarkers, and CT-based pancreatitis severity grading. Such multimodal integration may facilitate timely adjustments to care protocols and therapeutic interventions. Critically ill patients may benefit from these optimized strategies through improved functional outcomes and reduced mortality.

This research has multiple advantages. First, we investigated the connection between FI-lab and ICU patients suffering from acute pancreatitis for the first time. Second, we utilized the MIMIC-IV database as our data source, which lends a degree of credibility to our findings. Third, we demonstrated that FI-lab enhances the predictive capability of disease severity scoring systems concerning mortality. However, this research possesses certain limitations. Firstly, the retrospective nature of this study necessitated the exclusion of patients with incomplete data, which may introduce selection bias. Although multiple imputation was employed to address missing data, the baseline characteristics of excluded patients may differ from the analyzed cohort, potentially limiting the generalizability of findings. Secondly, the FI-lab score was derived solely from laboratory data collected within the first 24 h of ICU admission, failing to account for dynamic clinical changes that may influence prognosis, thereby introducing measurement bias. While temporal variations in FI-lab scores—reflecting evolving patient conditions—might hold greater clinical relevance, the relationship between dynamic FI-lab trajectories and outcomes in ICU-admitted acute pancreatitis patients remains undefined. Future prospective cohort studies are warranted to elucidate this association. Finally, the data were sourced from the MIMIC-IV database. Despite its strengths in scale and standardized data collection, the demographic homogeneity of the cohort (predominantly White individuals) and the advanced healthcare resource setting may limit the external validity of our conclusions, particularly when extrapolating to global populations in low-income regions or primary care facilities. Thus, caution is warranted in generalizing these findings. Further validation through multi-center, geographically diverse prospective studies is critical to assess the clinical utility of the FI-lab score across heterogeneous healthcare environments.

## 5 Conclusion

In this retrospective study of 741 ICU patients with acute pancreatitis, the FI-lab score independently predicted 28-day mortality and enhanced the prognostic accuracy of traditional severity scores. These findings support the integration of FI-lab into ICU risk stratification protocols to identify high-risk patients early. Future prospective studies should validate its utility across diverse populations and care settings.

## Data Availability

The original contributions presented in the study are included in the article/Supplementary material, further inquiries can be directed to the corresponding authors.
