# Supplementary material for: Relationship between the laboratory test-based frailty index and overall mortality in critically ill patients with acute pancreatitis: a retrospective study based on the MIMIC-IV database
Source: Front Med (Lausanne). 2025 Apr 8;12:1524358. doi: 10.3389/fmed.2025.1524358 (PMC12011769; doi:10.3389/fmed.2025.1524358)
Supplement: Supplementary file 1 [file Table_1.DOCX]

Supplementary Material

# Supplementary Tables

**TABEL S1 Reference range of items used for construction of FI-Lab**

| **Items** | **Reference range** |
| --- | --- |
| **Vital signs** |  |
| Systolic blood pressure (mm Hg) | 90–140 |
| Diastolic blood pressure (mm Hg) | 60–90 |
| Heart rate (bpm) | 60–99 |
| **Venous blood samples** |  |
| White cell count (×10^3^/μL) | 4–11 |
| Platelet count (×10^9^/L) | 150–440 |
| Hemoglobin (g/dL) | Female: 12–16 Male: 14–18 |
| Total bilirubin (mg/dL) | 0–1.5 |
| Alanine transaminase (Units/L) | 0–40 |
| Albumin (g/dL) | 3.5–5 |
| Alkaline phosphatase (Units/L) | 35–105 |
| Lactate dehydrogenase (Units/L) | 94–250 |
| Urea nitrogen (mg/dL) | 6–20 |
| Creatinine (mg/dL) | Female: 0.4–1.1 Male: 0.5–1.2 |
| Glucose (mg/dL) | 70–110 |
| Potassium (mmol/L) | 3.5–5.4 |
| Sodium (mmol/L) | 133–145 |
| Calcium (mg/dL) | 8.4–10.3 |
| Phosphorus (mg/dL) | 2.7–4.5 |
| Prothrombin time (s) | 9.4–12.5 |
| International normalized ratio | 0.9–1.1 |
| APTT (s) | 25–35 |
| Fibrinogen (mg/dL) | 150–400 |
| Troponin T (ng/mL) | 0–0.01 |
| **Arterial blood gas samples** |  |
| PH | 7.35–7.45 |
| PO_2_ (mm Hg) | 85–105 |
| PCO_2_ (mm Hg) | 35–45 |
| Lactate (mmol/L) | 0.5–2 |
| **Urine sample** |  |
| Leucocytes | Negative |
| Erythrocytes | Negative |
| Protein | Negative |
| Glucose | Negative |
| Ketones | Negative |
| Bilirubin | Negative |

1. Variables used in the calculation of the FI-lab score: This table lists the 33 variables used to calculate the FI-lab score, including three vital signs and 30 laboratory tests. For each variable, the reference range is provided. Variables exceeding the normal range were assigned a value of 1, while those within the normal range were assigned a value of 0. The FI-lab score was calculated by summing all assigned variable values and dividing by the total number of variables.
2. APTT activated partial thromboplastin time, PH potential of hydrogen, PO_2_ partial pressure of oxygen, PCO_2_ partial pressure of carbon dioxide.
3. Urine sample (Negative): The urine sample is not recorded in the MIMIC-IV database.
